# Th17 cells reflect colon submucosal pathologic changes in active eosinophilic granulomatosis with polyangiitis

**DOI:** 10.1186/s12865-015-0138-4

**Published:** 2015-12-29

**Authors:** Naomi Tsurikisawa, Chiyako Oshikata, Takahiro Tsuburai, Satoshi Sugano, Yoko Nakamura, Takuya Shimoda, Shunpei Tamama, Ken Adachi, Ayako Horita, Ikuo Saito, Hiroshi Saito

**Affiliations:** Departments of Allergy and Respirology, Sagamihara, Kanagawa Japan; Department of Medical Gastroenterology, Sagamihara, Kanagawa Japan; Department of Diagnostic Pathology, Sagamihara, Kanagawa Japan; Clinical Research Center for Allergy and Rheumatology, National Hospital Organization, Sagamihara National Hospital, 18-1 Sakuradai, Minami-ku, Sagamihara, Kanagawa 252-0392 Japan

**Keywords:** Churg-Strauss syndrome, Eosinophilic granulomatosis with polyangiitis, Intercellular adhesion molecule-1, Vascular cell adhesion molecule-1, Vascular endothelial growth factor

## Abstract

**Background:**

Chronic eosinophilic pneumonia (CEP) or eosinophilic gastroenteritis (EG), or both, with asthma precede the onset of eosinophilic granulomatosis with polyangiitis (EGPA) in half of all EGPA patients. It is not known what determines whether patients with CEP or with EG following asthma will develop EGPA.

**Methods:**

We studied 17 EGPA patients and 12 patients with CEP but without EGPA. We assayed serum ICAM-1, VCAM-1, and VEGF, and the percentage of peripheral blood CD4^+^ T cells producing IL-17 (Th17 cells), at both onset and remission. We also examined the numbers of submucosal eosinophils and the basement membrane-to-crypt and crypt-to-crypt distance to evaluate edema in the colon submucosa at onset and remission in EGPA and at onset in CEP.

**Results:**

Nine of 12 (75.0 %) CEP patients had symptoms or endoscopic findings. Colonic submucosal eosinophil counts and edema in EGPA at onset were greater than at remission or in CEP at onset. Th17 cells (%) and serum ICAM-1 levels at onset were greater in EGPA than in CEP. In EGPA, peripheral blood Th17 cells (%) were significantly correlated with serum ICAM-1 level, colonic submucosal eosinophil count, and degree of edematous change; inversely correlated with serum VEGF level; but not correlated with VCAM-1 level.

**Conclusions:**

Eosinophilia and colonic submucosal edematous change were greater in EGPA than in CEP. The mechanism of vasculitis in EGPA appears related to increases in serum Th17 cell numbers and ICAM-1 levels and decreases in VEGF levels.

## Background

Eosinophilic granulomatosis with polyangiitis (EGPA; formerly known as Churg-Strauss syndrome) is a rare disease characterized by allergic granulomatosis and necrotizing vasculitis developing after the appearance of peripheral and tissue eosinophilia [1]. Asthma is present in 96 to 100 % of EGPA patients and is the cardinal feature of EGPA. Asthma may precede systemic vasculitis by approximately 8 years and in some cases by more than 30 years [1–3]. Eosinophilic infiltrations, such as those found in chronic eosinophilic pneumonia (CEP) or eosinophilic gastroenteritis (EG), or both, precede systemic vasculitis in half of all patients with EGPA [3, 4]. Some patients with asthma complicated by CEP are likely to develop EGPA if the CEP is left untreated [5]. However, some patients in whom asthma is complicated by CEP do not develop EGPA. In our retrospective cohort study, we found that the clinical manifestations of asthma occurring in the pre-vasculitic phase of the disease were severe, or the percentage of eosinophils in the peripheral blood of pre-EGPA asthmatics at the first hospital visit was high, but bronchial hyperresponsiveness to acetylcholine in these pre-vasculitic patients was slight [6]. We subsequently confirmed that maintenance of regulatory T cell (T_reg_ cell) numbers in asthma patients with CEP may inhibit EGPA development via the action of cytokines, such as IL-10 and IL-2, produced by CD4^+^CD25^+^ and CD4^+^CD25^−^ T cells, respectively [7]. However, there is no clear mechanism that explains why some CEP patients with asthma develop EGPA and some do not.

Th17 cells play important roles in the immune response, depending on their stage of differentiation, be it induction, amplification, or stabilization. In mice, transforming growth factor beta (TGF-β) induces the development of inducible T_reg_ (iT_reg_) cells by inducing the expression of the transcription factor forkhead box P3 (Foxp3) and the production of interleukin (IL)-17A by CD4^+^ T cells. IL-6, IL-1β, tumor necrosis factor alpha, and IL-21, together with TGF-β, promote Th17 differentiation or polarization [8]. Some reports have suggested that Th17 cells are associated with vasculitis, as occurs in giant cell arteritis [9], Henoch-Schönlein purpura [10], antineutrophil cytoplasmic antibody (ANCA)-associated vasculitis [11], granulomatosis polyangiitis or Wegener’s granulomatosis [12], and EGPA [13, 14].

Intercellular adhesion molecule-1 (ICAM-1) and vascular cell adhesion molecule (VCAM)-1 are expressed predominantly on the surfaces of endothelial cells and play an essential role in local leukocyte recruitment to the vessel wall, adhesion of leukocytes to the endothelium, migration, and extravasation [15, 16]. An enhanced level of soluble VCAM-1 (sVCAM-1) is a marker of endothelial cell activation in ANCA-associated vasculitides [15]. Surface expression of ICAM-1, CD11b, and CD69 on eosinophils in the peripheral blood is increased in EGPA patients [17]. Vascular endothelial growth factor (VEGF) is secreted by endothelial cells and pericytes in response to hypoxia. It induces angiogenesis and microvascular hyperpermeability [18]. Serum VEGF levels are associated with disease activity, for example in ANCA-associated vasculitis [19] and systemic vasculitis [20].

Gastrointestinal tract involvement occurs in 17 to 92 % of patients with EGPA [1, 2, 21]. Characteristic symptoms in EGPA patients are abdominal pain, nausea or vomiting, diarrhea, hematochezia or melena, and hematemesis. Gastroduodenal ulceration has been detected endoscopically in 17 (27 %) of 62 patients with systemic vasculitis (SV) and colorectal ulceration in 6 (10 %), but in one study histologic signs of necrotizing vasculitis or granuloma could not be found in a number of small specimen [22]. Patients with EGPA who have severe gastrointestinal tract involvement, such as surgical abdomen, can develop peritonitis, bowel perforation, gastrointestinal ischemia or infarction, and intestinal occlusion, which carry a poor prognosis; mesenteric vasculitis may occur, leading to gastrointestinal ulceration, ischemia, and perforation [23]. Five-year survival rates in surgical patients with SV (EGPA patients were 11 of 62 patients [22], 82 of 342 patients [24]) have ranged from 41 to 56 % [22, 24]. Colonoscopic findings include mucosal erythema [23] and ulceration [25]. However, in EGPA specially, the incidence of another minor endoscopic signs such as red flare, and edematous change is not known. Moreover, to our knowledge, there have been no published investigations of the pathology of necrotizing vasculitis with infiltration other than eosinophils or granuloma. There were various eosinophilic gastrointestinal diseases shown by drugs or parasitic infection, inflammatory bowel disease, reflux esophagitis, coeliac disease, microscopic and infectious colitis, SV (including EGPA), and polyarteritis nodosa [6, 26, 27]. It is not known whether the intestinal endoscopic findings characteristics of EG in the asthmatic phase precede SV in patients with EGPA. Moreover, the pathological differences in the colon submucosa between patients with EGPA and those with CEP or other types of EG have not yet been investigated.

Here, we examined the pathological differences related to numbers of eosinophils and amount of edema in the colon submucosa between patients with EGPA and those with CEP. We also analyzed the relationship between pathological features and adhesion molecule or growth factor expression and the Th17 response.

## Results

### Clinical findings and treatment

There was no significant difference between patients with EGPA and those with CEP in terms of sex, atopic or non-atopic, presence or absence of allergic rhinitis or atopic dermatitis, age at time of onset of asthma, severity of asthma, and daily inhaled corticosteroid dose for treatment of asthma (Table 1). In five of 12 patients with CEP, eosinophilic pneumonitis improved without the patient taking systemic corticosteroids (i.e. prednisolone). However, all patients with EGPA received systemic corticosteroids (*P* < 0.01). The initial dose of prednisolone and the proportion of patients who used immunosuppressants or intravenous immunoglobulin (IVIG) were significantly higher in patients with EGPA than those in patients with CEP (Table 1; all *P* < 0.01). Our analysis of organ involvement revealed no significant differences between the two groups in terms of the rates of respiratory system involvement (asthma, paranasal sinusitis, and pulmonary infiltration) and the rates of involvement of the liver, gall bladder, and pancreas. EGPA patients had significantly higher rates of multiple polyneuropathy and myocardial, renal, skin, muscle, joint, and CNS involvement than CEP patients (*P* < 0.01 or *P* < 0.05).

**Table 1:** Characteristics and therapies of patients with eosinophilic granulomatosis with polyangiitis (EGPA) or chronic eosinophilic pneumonia (CEP)

	EGPA patients (*n* = 17)	CEP patients (*n* = 12)	*P*
Age (y), mean ± 1 SD	41.8 ± 15.9	44.2 ± 17.0	NS^a^
Sex (M/F)	7/10	6/6	NS^b^
Type: atopy/nonatopy	8/9	4/8	NS^b^
Allergic rhinitis (yes/no)	9/7	7/5	NS^b^
Atopic dermatitis (yes/no)	4/13	2/10	NS^b^
Age at onset asthma (y), mean ± SD	34.5 ± 14.3	36.3 ± 14.4	NS^a^
Astham severity Step 1/2/3/4	1/0/5/11	2/0/3/8	NS^b^
Daily dose of ICS (mg; converted to CFC-BDP equivalents)	1482.4 ± 656.0	1173.3 ± 812.3	NS^a^
Organ involvement at onset of EGPA or CEP patients (%)			
Asthma	100	100	NS^b^
Paranasal sinusitis	94.1	83.3	NS^b^
Multiple polyneuropathy	100	0	<0.01^b^
Pulmonary infiltrates	88.2	100	NS^b^
Myocardinal involvement	76.5	0	<0.01^b^
Liver, gall bladder, pancreas	17.6	0	NS^b^
Renal involvement^c^	41.2	0	< 0.05^b^
Proteinuria	41.2	0	< 0.05^b^
Nephritis or nephrosis	11.8	0	NS^b^
Skin involvement	88.2	25	< 0.01^b^
Arthritis	52.9	0	< 0.01^b^
Myalgia	29.4	0	< 0.05^b^
Central nervous system involvement	29.4	0	< 0.05^b^
Gastrointestinal tract			
Clinical symptoms on the upper abdominal region (yes/no)	10/7	3/9	NS^b^
Clinical symptoms on the lower abdominal region (yes/no)	12/5	8/4	NS^b^
Positive signs on mucous membrane of the upper digestive organs by stomach endscope (yes/no)	10/7	2/10	< 0.05^b^
Positive signs on mucous membrane of the large intestine by colon endscope (yes/no)	8/9	5/7	NS^b^
Treatment for EGPA or CEP			
Systemic corticosteroids (yes/no)	17/0	5/7	< 0.01^b^
Prednisolone per day for initial dose (mg), mean ± 1 SD	45.5 ± 10.7	20.2 ± 5.5	< 0.01^a^
Patients taking an immunosuppressant (%)	76.5	0	< 0.01^b^
CYC/AZA/CSA	6/3/4	0/0/0	
IVIG (yes/no)	9/8	0/12	< 0.01^b^

*AZA* azathioprine, *CFC-BDP* chlorofluorocarbon-propelled beclomethasone dipropionate, *CSA* ciclosporin, *CYC* cyclophosphamide, *ICS* inhaled corticosteroid, *IVIG* intravenous immunoglobulin, *NS* not significant

All values are expressed as means ± 1 SD

Values of *P* < 0.05 were considered statistically significant

^a^Two-way ANOVA employing a repeated-measures test to explore the significance of differences between any two groups

^b^Chi-squared testing revealed no significant differences in frequency between the two groups

^c^Renal involvement including protein uria or eosinophils in urin or glomerular nephritis or nephrosis or renal dysfunction

Asthma severity was according to GINA guide lines

### Gastrointestinal tract characteristics in EGPA and CEP patients

**Fig. 1:**
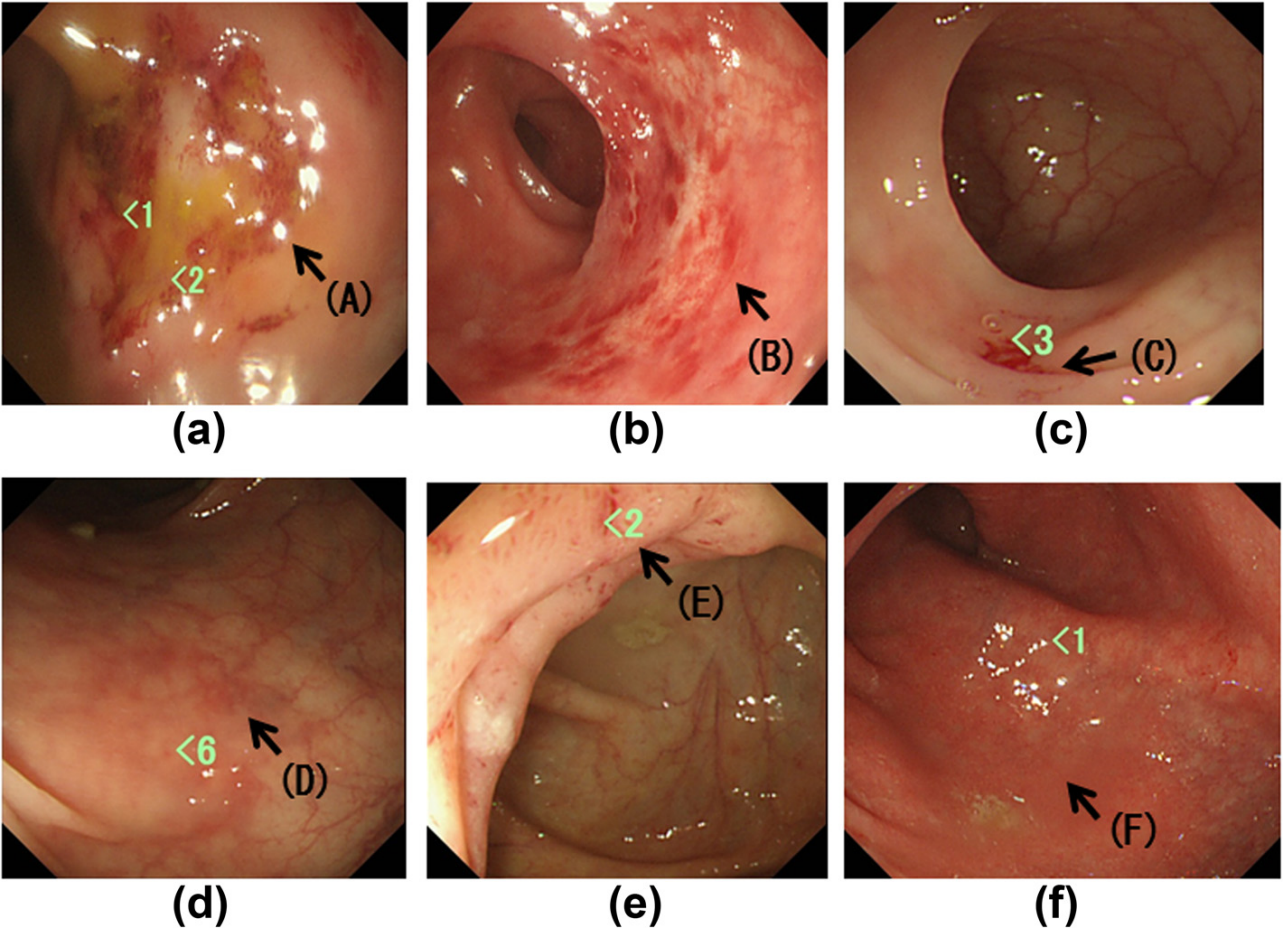
Endoscopic findings in the large intestine in patients with eosinophilic granulomatosis with polyangiitis (EGPA) (**a**–**c**) or chronic eosinophilic pneumonia (CEP) (**d**–**f**). Arrows show ulcer (*A*), erosion (*B*), dark red sign (*C*), and various red flares (*D*, *E*, *F*). The number in bright green indicated a region biopsied. EGPA patients had subjective symptoms related to the endoscopic findings in the lower digestive organs (e.g. abdominal pain, diarrhea, and blood in the stool), but there were no subjective symptoms in patients with CEP

The incidence of clinical symptoms related to both the upper and lower abdomen did not differ between the two groups (Table 1). There were various findings in the colonic mucosa (Fig. 1) as well as the gastro-duodenum mucosa (data not shown) in EGPA patients (ulceration, erosion, or dark red signs). In contrast, there were only slight changes in the colonic mucosa, such as red flare, in patients with CEP (and Fig. 1). However, the overall incidence of positive colonic mucosal findings on endoscopy did not differ between the two groups. In contrast, positive gastroscopic findings were more common in EGPA patients than those in CEP patients (*P* < 0.05, Table 1).

### Counts and percentages of white blood cells (WBCs), eosinophils, and Th17 cells, and serum ICAM-1, VCAM-1, and VEGF levels

The numbers of WBCs and eosinophils in the peripheral blood at disease onset were significantly higher in EGPA patients than in CEP patients (*P* < 0.05 and *P* < 0.01, respectively; Table 2). Serum IgE radioimmunosorbent assay values did not differ between the two groups.

**Table 2:** Comparison of peripheral blood biomarkers or pathological findings in the large intestinal mucosa between patients with eosinophilic granulomatosis with polyangiitis (EGPA) and those with chronic eosinophilic pneumonia (CEP)

	EGPA patients (*n* = 17)		Comparison between EGPA patients with at onset and at remission	CEP patients (*n* = 12)		Comparison between CEP patients with at onset and after an improvement
	At onset	At remission	*P*	At onset	After an improvement	*P*
Peripheral blood						
WBC (/mL) in blood, mean ± 1 SD	15,150 ± 9,062^b,a^	8,655 ± 2,542	< 0.01^a^	9,906 ± 4,637	6,928 ± 2,086	< 0.01^b^
Blood eosinophils (/mL), mean ± 1 SD	7,356 ± 6,293^b,**^	142.9 ± 132.7	< 0.01^a^	3,390 ± 3,575	585.0 ± 443.0	< 0.01^a^
log IgE RIST in serum	2.910 ± 0.495	0.754 ± 0.259	< 0.01^a^	2.647 ± 0.505	0.921 ± 0.445	< 0.01^a^
MPO-ANCA (%)	41.2^c,*^	0	< 0.05^c,*^	0	0	NS^c^
PR3-ANCA (%)	0	0	NS^c^	0	0	NS^c^
ICAM-1 level in serum (ng/mL)	531.5 ± 136.4^b,*^	392.2 ± 128.3	< 0.01^a^	372.0 ± 259.6	343.4 ± 37.3	NS^a^
VCAM-1 level in serum (ng/mL)	2633.6 ± 4102.7	756.5 ± 563.9	< 0.05^a^	651.6 ± 132.0	680.2 ± 415.1	NS^a^
VEGF level in serum (pg/mL)	231.9 ± 169.4	388.7 ± 265.3	< 0.05^a^	192.1 ± 99.1	104.8 ± 111.0	NS^a^
Percentage of CD4^+^ T cells producing IL-17 (%)	8.9 ± 5.2^b,**^	0.7 ± 1.8	< 0.01	2.2 ± 2.7	1.0 ± 1.8	NS^a^
Pathological findings in colon						
Necrotizing vasculitis (yes/no)	0/17	0/17	NS^c^	0/12	N.D	NS^c^
Granuloma (yes/no)	0/17	0/17	NS^c^	0/12	N.D	NS^c^
The number of eosinophils in submucosa (/mm^2^)	113.0 ± 46.6^b,*^	29.5 ± 18.8	< 0.01^a^	81.9 ± 49.3	N.D	
Interval between basement membrane and crypt (/mm)	7.7 ± 1.3^b,**^	5.8 ± 1.2	< 0.01^a^	5.4 ± 1.9	N.D	
Interval between crypt and crypt (/mm)	5.5 ± 1.0^b,**^	3.7 ± 1.7	< 0.01^a^	2.8 ± 1.0	N.D	

*ANCA* antineutrophil cytoplasmic antibodies, *ICAM-1* intercellular adhesion molecule-1, *MPO* myeloperoxidase, *NS* not significant, *PR3* proteinase 3, *RIST* radioimmunosorbent, *VCAM-1* vascular cell adhesion molecule-1, *VEGF* vascular endothelial growth factor, *WBCs* white blood cells

All values are expressed as means ± SD

^a^Statistical comparisons performed with the Wilcoxon matched-pairs *T*-test

^b^Two-way ANOVA with repeated measures between groups

^c^Chi-squared testing revealed no significant differences between groups

Values of *P* < 0.05 were considered statistically significant

^*^EGPA at onset vs. CEP at onset, *P* < 0.05

^**^EGPA at onset vs. CEP at onset, *P* < 0.01

None of the CEP patients was positive for MPO- or PR3-ANCA. The serum ICAM-1 level at onset was greater in EGPA patients than in CEP patients (*P* < 0.05), but this was not the case for the serum VCAM-1 and VEGF levels. In EGPA patients, serum ICAM-1, VCAM-1, and VEGF levels were significantly higher at onset than at remission, but those in CEP patients did not change between onset and improvement. The percentage of CD4^+^ T cells producing IL-17 (so-called Th17 cells) at disease onset was significantly higher in patients with EGPA than in those with CEP (*P* < 0.01). The percentage of Th17 cells in EGPA patients was significantly lower at remission than at disease onset (*P* < 0.01), but there was no change in this percentage in CEP patients between onset and improvement (Table 2).

### Pathological findings in the colon

**Fig. 2:**
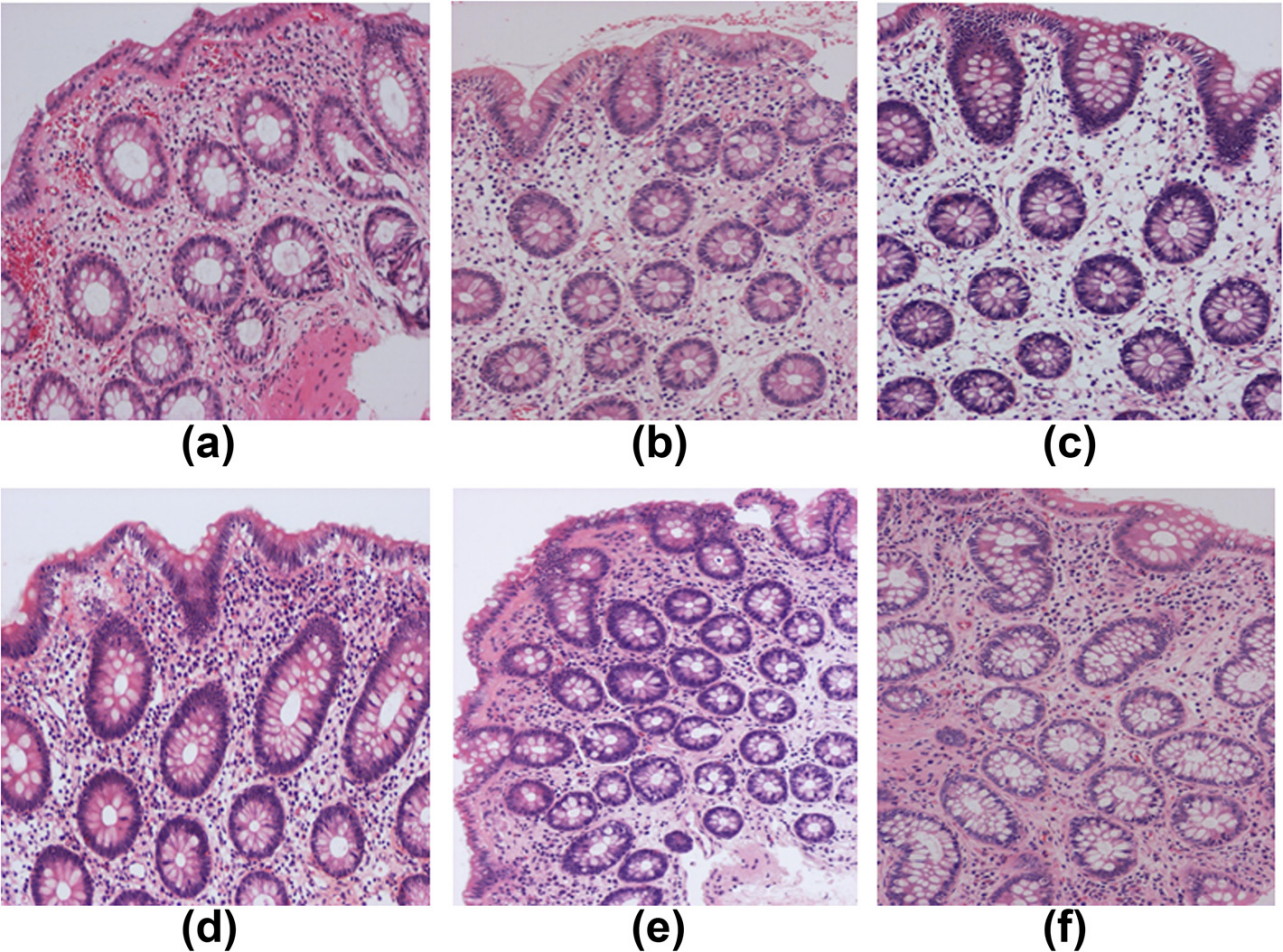
Pathological findings in the large intestine in patients with eosinophilic granulomatosis with polyangiitis (EGPA) (**a**–**c**) or chronic eosinophilic pneumonia (CEP) (**d**–**f**). Evidence of bleeding was present in the submucosa (**a**). The number of eosinophils in the submucosa was higher in patients with EGPA (**a**–**c**) than in those with CEP (**d**–**f**). Edematous changes (expressed as the width of the basement membrane-to-crypt distance or the crypt-to-crypt distance) were more severe in patients with EGPA than in those with CEP

**Fig. 3:**
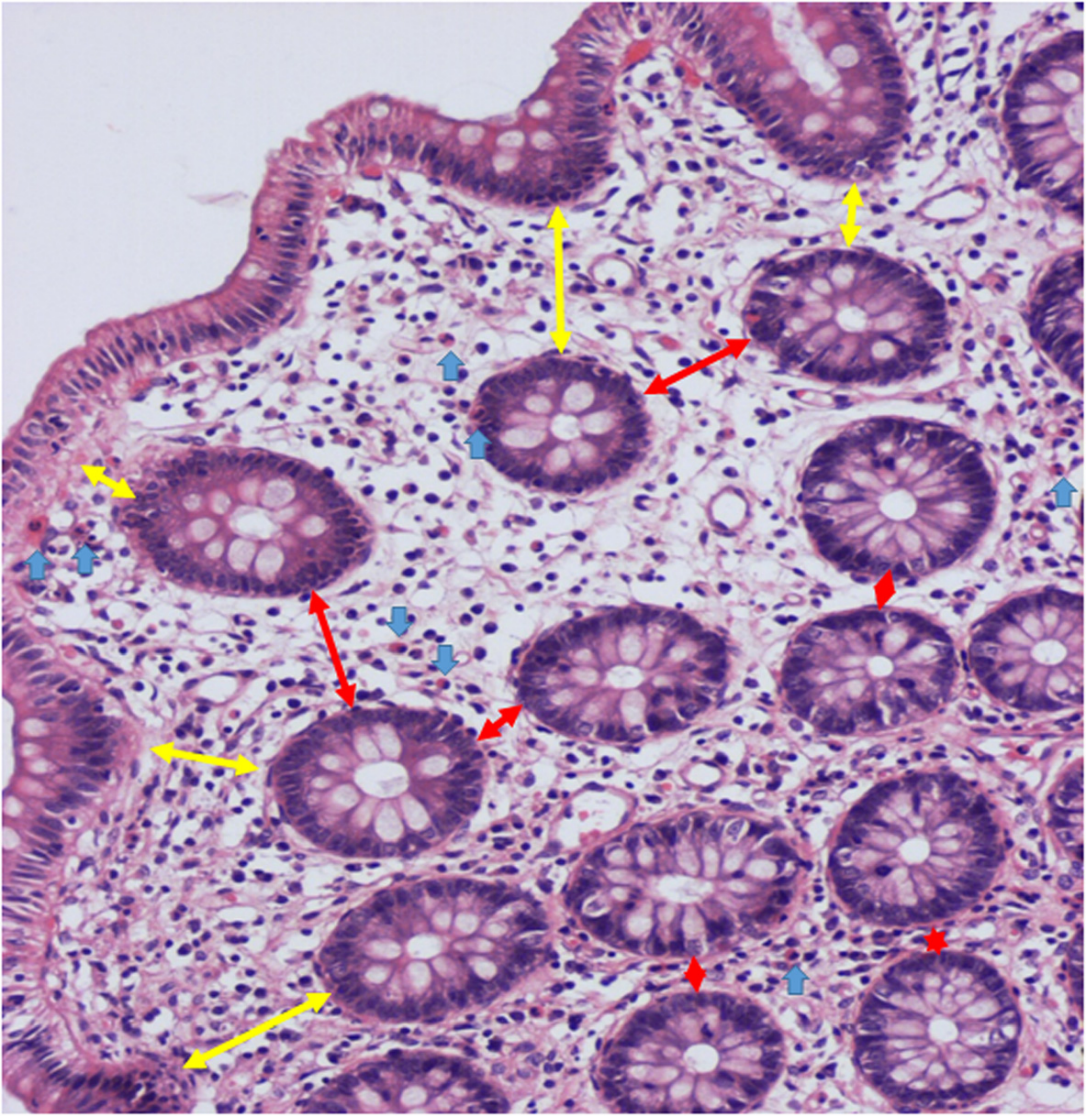
Assessment of number of eosinophils and edema in colonic submucosa in a pathology specimen from a patient with eosinophilic granulomatosis with polyangiitis. Blue arrows indicate eosinophils. Red arrows show crypt-to-crypt distance. Yellow arrows show basement membrane-to-crypt distance. All values are expressed as means ± SD in Table 2

We could not find any necrotizing vasculitis or granuloma in small size samples of submucosa from patients with either EGPA or CEP. At disease onset there were significantly more eosinophils in the colonic submucosa of EGPA patients than of CEP patients (*P* < 0.05) (Table 2, Fig. 2). Submucosal eosinophil counts in EGPA were significantly lower at remission than at onset (*P* < 0.05). The localization of submucosal edema varied within each patient (Fig. 3). The basement membrane-to-crypt interval and crypt-to-crypt distance reflected the degree of edema in the colonic submucosa. The basement membrane-to-crypt and crypt-to-crypt distance at disease onset were significantly greater in EGPA patients than in CEP patients (*P* < 0.01) (Fig. 2). In CEP patients, there were few, if any, edematous changes in the submucosa. The basement membrane-to-crypt and crypt-to-crypt distance in EGPA patients were significantly shorter at remission than at onset (*P* < 0.01) (Table 2).

### Correlation between eosinophil counts in peripheral blood and colonic submucosa in patients with EGPA or CEP

**Fig. 4:**
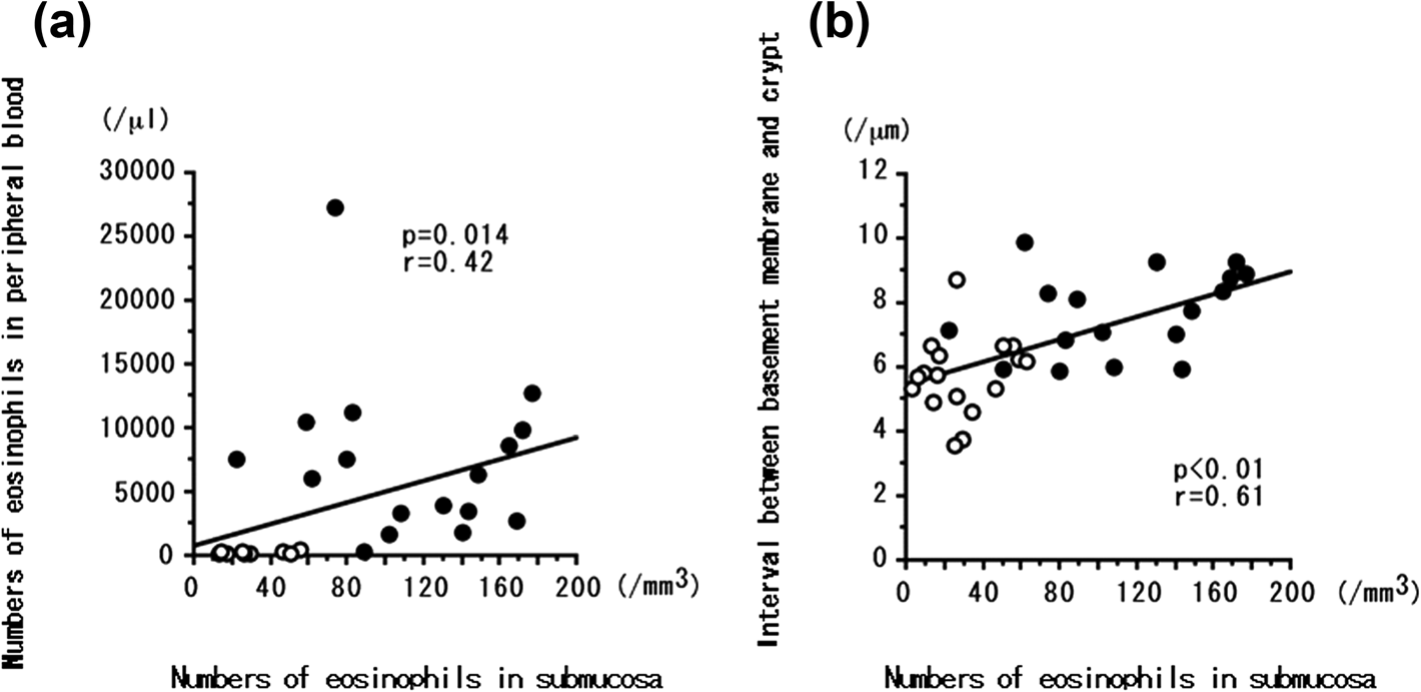
Correlations between eosinophils count (per microliter) in the colonic submucosa and eosinophil count in the peripheral blood (**a**) or the basement membrane-to-crypt distance (**b**) in patients with eosinophilic granulomatosis with polyangiitis (EGPA). The basement membrane-to-crypt distance reflected edema in the colonic submucosa. *Closed circles*: mucous membrane of colon biopsied at the onset of EGPA. *Open circles*: mucous membrane of colon biopsied in remission after treatment with corticosteroids with or without immunosuppressants. Correlation coefficients (*r* values) and *P* values were calculated by using Spearman’s rank correlation test

The number of peripheral blood eosinophils in EGPA patients at both onset and remission was significantly correlated with the eosinophil count in the submucosa (*P* = 0.014, *r* = 0.42) (Fig. 4a), but this was not the case in CEP patients at onset (*P* = 0.42, *r* = 0.26) (data not shown).

### Correlation between eosinophil count in colonic submucosa and basement membrane-to-crypt interval in patients with EGPA or CEP

The number of eosinophils in the colonic submucosa of EGPA patients at both onset and remission was significantly correlated with the basement membrane-to-crypt distance (*P* < 0.01, *r* = 0.61) (Fig. 4b), but this was not the case in CEP patients at onset (*P* = 0.37, *r* = –0.29) (data not shown). Similarly, the number of eosinophils in the colonic submucosa of EGPA patients was significantly correlated with the crypt-to-crypt distance (*P* < 0.01, *r* = 0.53), but this was not the case in CEP patients at disease (data not shown). The number of eosinophils in the peripheral blood of EGPA patients at onset and remission was significantly correlated with the crypt-to-crypt distance (*P* < 0.01, *r* = 0.52), but again this was not the case in CEP patients at disease onset (data not shown).

### Correlations between percentage of Th17 cells in peripheral blood and eosinophil count in submucosa, crypt-to-crypt interval, and basement membrane-to-crypt interval in patients with EGPA

**Fig. 5:**
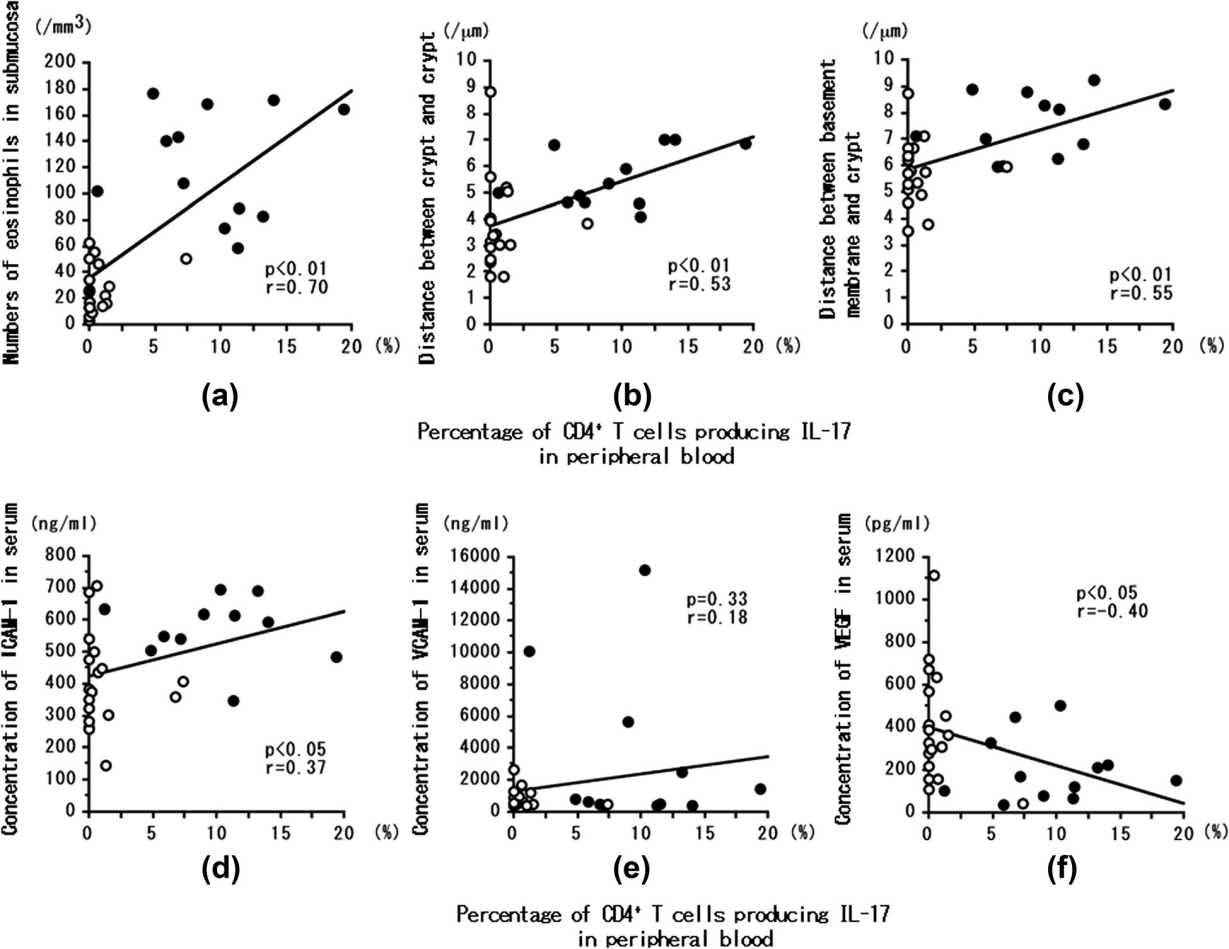
Correlations between the percentage of CD4^+^ T cells producing IL-17 in peripheral blood and eosinophil count in the colonic submucosa (**a**); the crypt-to-crypt distance (**b**); the basement membrane-to-crypt distance (**c**), the ICAM-1 level (**d**); the VCAM-1 level (**e**); and the VEGF level (**f**) in the sera of patients with eosinophilic granulomatosis with polyangiitis (EGPA). *Closed circles*: mucous membrane of colon biopsied at onset of EGPA. *Open circles*: mucous membrane of colon biopsied in remission after treatment with corticosteroids with or without immunosuppressants. Correlation coefficients (*r* values) and *P* values were calculated by using Spearman’s rank correlation test

The percentage of Th17 cells in the peripheral blood of EGPA patients at onset and remission was significantly correlated with the submucosal eosinophil count (*P* < 0.01, *r* = 0.70) (Fig. 5a), with the crypt-to-crypt distance (*P* < 0.01, *r* = 0.53) (Fig. 5b), and with the basement membrane-to-crypt distance (*P* < 0.01, *r* = 0.55) (Fig. 5c).

### Correlations between percentage of Th17 cells in peripheral blood and serum ICAM-1, VCAM-1, and VEGF levels in patients with EGPA

The percentage of Th17 cells in the peripheral blood of EGPA patients at onset and remission was significantly positively correlated with the serum ICAM-1 level (*P* < 0.05, *r* = 0.37) (Fig. 5d) and inversely correlated with the serum VEGF level (*P* < 0.05, *r* = – 0.40) (Fig. 5f), but it was not correlated with the serum VCAM-1 level (NS, *r* = 0.18) (Fig. 5e). The number of submucosal eosinophils was significantly correlated with the serum ICAM-1 level (*P* < 0.01, *r* = 0.44), but not with the serum VCAM-1 or VEGF level (data not shown). The number of eosinophils in the peripheral blood was significantly correlated with the serum ICAM-1 level (*P* < 0.05, *r* = 0.39) and VCAM-1 level (*P* < 0.01, *r* = 0.67), but not with the serum VEGF level (data not shown). The basement membrane-to-crypt distance was significantly correlated with the serum ICAM-1 level (*P* < 0.05, *r* = 0.44) but not with the serum VCAM-1 or VEGF level (data not shown).

## Discussion

The 5-year survival rate in EGPA is 62 to 97 % [21, 24, 28–30]. The prognosis of EGPA patients and the extent of mortality from the disease are associated with disease severity, as assessed by using the Five-Factor Scores developed in 1996 [24] and 2009 [29]. In addition, myocardial involvement [2] and the presence of gastrointestinal disease [2, 31] are independent negative prognostic factors. However, the gastrointestinal pathology of EGPA has not yet been fully characterized. Moreover, to our knowledge, differences in EG have not been investigated between patients with EGPA at disease onset and patients with asthma preceding the development of SV in the form of EGPA. Among the various eosinophilic gastrointestinal diseases [27, 32], there is less submucosal edema in ulcerative colitis, Crohn’s disease, and collagenous colitis [27, 33] than we saw here in our EGPA patients.

Until now there has been a lack of detailed information on pathological findings in the colonic submucosa. In our EGPA patients, we expected to find eosinophilia but were surprised to find no necrotizing vasculitis or granuloma. The presence of edematous change was also previously unreported. The eosinophil count in the submucosa was significantly correlated with the eosinophil count in the blood and with the basement membrane-to-crypt interval in patients with EGPA (Fig. 4), but not in patients with CEP (data not shown). The lack of correlation in the latter might have been caused by the fact that in these patients the colon was biopsied only once (at the onset of CEP), whereas in EGPA patients the colon was biopsied twice (at disease onset and at remission). All patients with asthma did not suffer eosinophilic gastrointestinal disorder. And there were an infiltration of eosinophils extravascular area in patients with asthma and without any gastrointestinal symptoms. Moreover there was an infiltration of number of eosinophils extra vascular extravascular tissue in patients with having more than 2052 number of eosinophils in peripheral blood [34].

There were some reports related to type 2 cytokine and EGPA [35, 36]. However there was little biomarkers distinguishing between EGPA and another hypereosinophilic syndrome [37].

We showed here that Th17 cell production decreased at remission in patients with EGPA. Previously, we showed that the count of iT_reg_ cells producing IL-10 was reduced in EGPA patients at disease onset and upon relapse, but increased when the disease was inactive [7]. Here, we investigated FOXP3^+^/CD4^+^ T cells as natural T_reg_ cells in patients with EGPA or CEP. We confirmed that the percentage of Th17 cells was inversely correlated with the number of FOXP3^+^/CD4^+^ T cells (*P* < 0.01, *r* = -0.57) (data not shown). The number of FOXP3^+^/CD4^+^ T cells was significantly correlated with the number of eosinophils in the colonic submucosa (*P* < 0.01, *r* = 0.59) (data not shown), but unlike the Th17 cell count it was not correlated with the interval between basement membrane and crypt or between crypt and crypt (data not shown). This result indicated that the Th17 cell count but not the Treg cell count reflected the occurrence of pathological changes related to vasculitis. After treatment of EGPA patients, the serum ICAM-1 and VCAM-1 levels decreased significantly, whereas the serum VEGF level increased significantly (Table 2). The serum ICAM-1 level was significantly correlated with the VCAM-1 level (*P* = 0.011, *r* = 0.40) (data not shown). We found previously that multiple polyneuropathy and cardiac dysfunction improved after IVIG administration [38], and the numbers of T_reg_ cells (as FOXP3^+^/CD4^+^ T cells or CD4^+^ T cells producing IL-10) increased for two years [39].

Maternal sera obtained after IVIG treatment, and polyclonal IVIG, decrease constitutive and cytokine-induced ICAM-1 and VCAM-1 expression on vascular endothelial cells ex vivo [40]. The combination of IVIG and methylprednisolone generally has a greater suppressive effect on mRNA expression and on the production of VCAM-1, IL-1β, and VEGF on endothelial cells derived from human umbilical vein [41]. IVIG-induced inhibition of angiogenesis has an inhibitory effect on VEGF-mediated blood perfusion in the ischemic limb in a mouse model [42]. However, the positive clinical effects of high-dose IVIG on muscle function in patients with refractory inflammatory active myositis in the form of polymyositis or dermatomyositis are not accompanied by any effects on ICAM-1 and VCAM-1 [43]. We considered that treatment with various combinations of corticosteroids, immunosuppressants, and IVIG induced clinical remission, decreased ICAM-1 and VCAM-1 levels, and increased VEGF levels. The sICAM-1 level was high in EGPA patients at disease onset, but the sVCAM-1 level varied widely and was relatively high at the time of onset of EGPA. An increase in Th17 cell numbers likely reflects active vasculitis [14], and an increase in the number of eosinophils or WBCs might induce upregulation of the expression of ICAM-1 or VCAM-1 on vascular endothelial cells; inflammatory cells such as eosinophils and lymphocytes, including Th17 cells, then migrate extravascularly into the colonic submucosa. An increase in serum VEGF level might have contributed to neovascularization.

We showed the correlation with Th17 cells in peripheral blood and ICAM-1 or VEGF in serum. We speculated the decrease of the number of Th17 cells after the treatment of steroid and immunosuppressant might affect the decrease of ICAM-1 and the increase of VEGF. These results were reflected to an inhibition of migration inflammatory cells and to promote neovascularization.

## Conclusion

In summary, we clarified the pathological and immunological differences between patients with EGPA and those with CEP. We expect that this clarification will help us to diagnose EGPA in the early stages and to start treatment early, thus giving patients the chance of a good prognosis.

## Methods

### Patients

Between April 2008 and October 2012, we recruited 17 patients with EGPA and 12 patients with CEP but without EGPA. All patients with EGPA and CEP were recruited at the Clinical Research Center for Allergy and Rheumatology, National Hospital Organization, Sagamihara, Kanagawa, Japan. EGPA was defined according to the classification criteria of the American College of Rheumatology [44]. Patients with CEP were diagnosed with asthma by using the criteria of the Global Initiative for Asthma; the severity of asthma was also rated with the aid of these guidelines [45]. CEP was defined as the fulfillment of at least two of three criteria, namely respiratory symptoms of more than 2 weeks’ duration; alveolar or blood eosinophilia (alveolar eosinophilia >25 % on bronchoalveolar lavage fluid differential cell count; blood eosinophilia >1000/mm^3^); and pulmonary infiltrates with a usually peripheral predominance on chest imaging [46]. Patients with CEP who had allergic bronchopulmonary mycosis were excluded from the study.

Multiple mononeuritis—a measure of motor nerve dysfunction—was evaluated by using manual muscle testing; responses were scored from zero to five on the Medical Research Council scale. Sensory nerve dysfunction was evaluated by clinical examination. Lung involvement in EGPA patients was considered present when any of consolidation, ground grass opacity, nodules within such opacity, interlobular septal thickening, bronchial wall thickening, lymph node enlargement, pleural effusion evident upon high-resolution computer tomography, or eosinophil infiltration detected by lung biopsy was present. The heart was considered to be involved when any of chest pain, chest discomfort, back pain, palpitations, abnormal signs on cardiac echocardiography, Holter electrocardiographic abnormalities, elevated B-type natriuretic peptide levels, or [^123^I]-metaiodobenzylguanidine myocardial imaging abnormalities of the myocardium was evident [47]. Gastrointestinal involvement was indicated by the presence of symptoms of epigastralgia, abdominal pain, diarrhea, or constipation, or of positive endoscopic signs, combined with confirmation of eosinophil infiltration by biopsy. Skin involvement was defined as the presence of purpura, erythema, livedo, or ulceration, or the presence of acrocyanosis when a nodule, accompanied by eosinophilic infiltration, was additionally detected by biopsy. Central nervous system involvement was defined as the presence of headache, visual disorder, abnormal visual sensation, cerebral infarction, bleeding, or cranial nerve dysfunction. Renal involvement was defined by any of the presence of eosinophils in the urine, glomerular nephritis, nephrosis, renal dysfunction (i.e., creatinine level >20 % above baseline), or proteinuria (>0.5 g/day).

All patients with EGPA were treated with conventional therapy (corticosteroids, immunosuppressants, or both; the initial dose of corticosteroid was approximately 1 mg/kg prednisolone daily for at least 1 month) [48]. Remission was defined as the absence of any clinical signs or symptoms of active vasculitis for at least 6 months after these treatments. Patients with CEP who had not entered spontaneous remission received treatment with systemic corticosteroids in addition to inhaled corticosteroids.

At disease onset and at remission in patients with EGPA, and at disease onset and improvement in patients with CEP, we examined the whole blood cell count; eosinophil count in whole blood; serum IgE concentration; serum MPO- or PR3-ANCA level and ICAM-1, VCAM-1, and VEGF levels; and the percentage of CD4^+^ T cells producing IL-17 in the peripheral blood. Endoscopy of the large intestine was performed at onset and remission in patients with EGPA, and at onset in patients with CEP. The Ethics Committee of our NHO Sagamihara hospital approved the study, and written informed consent was obtained from each patient.

### Endoscopy of the large intestine or the gastro-duodenum and biopsy of the colon mucosa

Endoscopy of the large intestine was performed after irrigation of the colon with 68 g of magnesium citrate dissolved in 1800 mL of water; each patient was anesthetized intravenously with 3 mg midazolam hydrochloride [49]. A colonscope (Olympus CF-H290I, Olympus Optical Co., Tokyo, Japan) was inserted through the rectum and up into the sigmoid colon, ascending colon, transverse colon, descending colon, and ileocecum. Endoscopy of the gastro-duodenum was performed with using gastro fiberscope (Olympus GIF-XP260N, Olympus Optical Co., Tokyo, Japan). At least one point (the gastro-duodenum) or three points (the large intestine) on the mucous membrane were biopsied from both visible points and invisible findings.

### Measurement of basement membrane-to-crypt interval, crypt-to-crypt interval, and numbers of eosinophils in submucosa

Biopsy specimens were fixed in 10 % formaldehyde, embedded in paraffin, cut at 3 μm, and stained with hematoxylin and eosin. To our knowledge, there is no accepted method for measuring the amount of edema in the colonic submucosa. We therefore chose to assess the grade of edema in the submucosa in accordance with the method used in a report by Sanderson et al. [50].

The crypt-to-crypt distance in the submucosa was measured as the closest distance between two crypts at 10 or more points in randomly selected fields of the biopsy specimen (Fig. 3). In the same way, the basement membrane-to-crypt distance was measured as the closest distance between basement membrane and crypt at 10 or more points. Similarly, the numbers of eosinophils in the submucosa were counted at five points or more in randomly selected fields of the biopsy specimen (Fig. 3). We used image analysis software (Image Pro Plus 6.0, Media Cybernetics, MD, USA) to evaluate edema and eosinophilic inflammation in the colonic submucosa. To determine the number of eosinophils, we counted the number of cells per slide and then converted that number to number of eosinophils/mm^2^. All values are expressed as means ± SD.

### Immunological analysis

#### Induction of cytokine expression and staining of intracellular cytokines

Fluorescein isothiocyanate–bearing conjugates of mouse IgG1 and anti-CD4, and phycoerythrin (PE)-conjugated anti-human IL-17 were purchased from BioLegend or R&D Systems (Cosmo Bio Co. Ltd., Tokyo, Japan). To induce cytokine expression and accumulation, peripheral blood mononuclear cells (PBMCs, 1 × 10^6^ cells/mL) suspended in RPMI 1640 medium supplemented with 10 % Fetal Calf Serum (FCS) were stimulated for 4 h at 37 °C with 10 μg/mL brefeldin A in the presence or absence of 50 ng/mL phorbol myristate acetate (PMA) and 1 μg/mL ionomycin [51]. Cells that died after this stimulation were removed. The percentage of dead cells among PBMCs treated with PMA and ionomycin was calculated by exclusion-testing by staining with 0.4 % trypan blue solution. As a control we used whole blood cells diluted to the same extent in FCS-free RPMI 1640 before the addition of trypan blue. The percentage of dead cells was 6.8 ± 6.1 %. Dead cells stained with trypan blue could not be distinguished in the fluorescence-activated cell sorting (FACS). However, even if these dead cells were included, the statistical significance of the percentage of positive cells producing cytokines was unaffected.

Surface-stained whole-blood lymphocyte samples were suspended in 0.5 mL cold 4 % (v/v) paraformaldehyde (used as a fixative) and incubated at room temperature for 10 min. Next, the cells were washed twice with phosphate-buffered saline (PBS) and centrifuged at 200 *g* for 7 min. Each pellet thus obtained was suspended in 2 mL serum amyloid P component (SAP) buffer (0.1 % [w/v] saponin, 0.05 % [w/v] NaN_3_, in Hanks’s balanced salt solution). The cell suspension was again centrifuged at 200 *g* for 7 min, and the cell pellet was suspended in 0.1 mL SAP buffer. Cell suspensions were diluted with PBS and aliquoted into tubes containing 10^6^ cells/20 μL. Phycoerythrin-conjugated anti-IL-17 was added to each tube. All tubes were vortexed and incubated for 35 min at room temperature in the dark. The percentage of cells generating cytokines was measured with a FACSCalibur (Nippon Becton Dickinson, Tokyo, Japan) flow cytometry system and the data were analyzed with the aid of CELLQuest software (Nippon Becton Dickinson, Tokyo, Japan).

### Soluble ICAM-1, sVCAM-1, and VEGF levels in serum

Soluble ICAM-1 (sICAM-1), sVCAM-1, and VEGF levels in the serum were assayed by using Human soluble ICAM-1, VCAM-1, and VEGF Platinum ELISA kits (eBioscience, San Diego, CA, USA). For quantification of sICAM-1 and sVCAM-1, those plates were washed twice with 400 μg of wash buffer (PBS with 0.05 % Tween 20). Then 100 μL of Assay Buffer and 100 μL of diluted plasma (in the case of VCAM-1) or 10 μL of sample (in the case of ICAM-1) was incubated in each plate well. Fifty micrograms of conjugate mixture (biotin-conjugated anti-human sICAM-1 or sVCAM-1) was added to the well, which was then incubated for 1 h (sICAM-1) or 2 h (sVCAM-1) at room temperature. After each well had been washed three times, 100 mL of tetramethylbenzidine (TMB) Substrate Solution was added to each well and the mixture was incubated for 10 min at room temperature. A stop solution (1 M phosphoric acid) was then added and the results analyzed by using an ELISA reader.

For quantification of VEGF, plates were first washed twice with 400 μg of wash buffer, then 50 μL of Assay Buffer and 50 μL of sample were incubated in each plate well for 2 h at room temperature. One hundred micrograms of conjugate mixture (biotin-conjugated anti-human VEGF-A) was added and the well was incubated for 1 h at room temperature. After the wells had been washed six times, 100 mL of TMB Substrate Solution was added to each well and the mixture incubated for 30 min at room temperature. After another six washings of the wells, VEGF levels were analyzed with an ELISA reader after addition of the stop solution as above.

### Statistical analysis

All values are expressed as means ± SDs unless otherwise specified. Statistical comparisons among groups were achieved by using two-way analysis of variance (ANOVA) employing a repeated-measures algorithm, followed by post-hoc comparisons using the Newman-Keuls test. Two mean values were compared by using the Mann-Whitney *U*-test. The two mean values obtained by this process were compared by using the Wilcoxon matched-pairs *T*-test. Correlation coefficients were obtained by employing Spearman’s rank correlation test. *P* values <0.05 were considered statistically significant. Statistical analysis was performed with SPSS for Windows, version 20 (SPSS Inc., Chicago, IL).

## Abbreviations

ANCA: Antineutrophil cytoplasmic antibody
CEP: Chronic eosinophilic pneumonia
EG: Eosinophilic gastroenteritis
EGPA: Eosinophilic granulomatosis with polyangiitis
FACS: Fluorescence-activated cell sorting
FOXP3: Forkhead box P3
ICAM-1: Intercellular adhesion molecule-1
MPO: Myeloperoxidase
PMA: Phorbol myristate acetate
PMBC: Peripheral blood mononuclear cell
PBS: Phosphate-buffered saline
PR3: Proteinase 3
SAP: Serum amyloid P
T_reg_ cell: Regulatory T cell
sICAM-1: Soluble intercellular adhesion molecule-1
SV: Systemic vasculitis
sVCAM-1: Soluble vascular cell adhesion molecule-1
TGF-β: Transforming growth factor beta
VCAM-1: Vascular cell adhesion molecule-1
VEGF: Vascular endothelial growth factor
WBC: White blood cell

## References

[1] Churg J, Strauss L (1951). Allergic granulomatosis, allergic angiitis, and periarteritis nodosa. Am J Pathol.

[2] Guillevin L, Cohen P, Casassus P, Lhote F, Jarrousse B, Casassus P (1999). Churg-Strauss syndrome. Clinical study and long-term follow-up of 96 patients. Medicine.

[3] Lhote F, Guillevin L (1995). Polyarteritis nodosa, microscopic polyangiitis, and Churg-Strauss syndrome. Clinical aspects and treatment. Rheum Dis Clin North Am.

[4] Steinfeld S, Golstein M, De Vuyst P (1994). Chronic eosinophilic pneumonia (CEP) as a presenting feature of Churg-Strauss syndrome (CSS). Eur Respir J.

[5] Churg A (2001). Recent advances in the diagnosis of Churg-Strauss syndrome. Mod Pathol.

[6] Tsurikisawa N, Tsuburai T, Saito H, Morita S, Horiguchi Y, Mitomi H (2007). A retrospective study of bronchial hyperresponsiveness in asthmatic patients prior to the onset of Churg-Strauss Syndrome. Allergy Asthma Proc.

[7] Tsurikisawa N, Saito H, Tsuburai T, Oshikata C, Ono E, Mitomi H (2008). Differences in regulatory T cells between Churg-Strauss syndrome and chronic eosinophilic pneumonia with asthma. J Allergy Clin Immunol.

[8] Nembrini C, Maeskand BJ, Kopf M (2009). IL-17-producing T cells in lung immunity and inflammation. J Allergy Clin Immunol.

[9] Terrier B, Geri G, Chaara W, Allenbach Y, Rosenzwajg M, Costedoat-Chalumeau N (2012). Interleukin-21 modulates Th1 and Th17 responses in giant cell arteritis. Arthritis Rheum.

[10] Jen HY, Chuang YH, Lin SC, Chiang BL, Yang YH (2011). Increased serum interleukin-17 and peripheral Th17 cells in children with acute Henoch-Schönlein purpura. Pediatr Allergy Immunol.

[11] Nogueira E, Hamour S, Sawant D, Henderson S, Mansfield N, Chavele KM (2010). Serum IL-17 and IL-23 levels and autoantigen-specific Th17 cells are elevated in patients with ANCA-associated vasculitis. Nephrol Dial Transplant.

[12] Abdulahad WH, Stegeman CA, Limburg PC, Kallenberg CG (2008). Skewed distribution of Th17 lymphocytes in patients with Wegener’s granulomatosis in remission. Arthritis Rheum.

[13] Jakiela B, Sanak M, Szczeklik W, Sokolowska B, Plutecka H, Mastalerz L (2011). Both Th2 and Th17 responses are involved in the pathogenesis of Churg-Strauss syndrome. Clin Exp Rheumatol.

[14] Saito H, Tsurikisawa N, Tsuburai T, Oshikata C, Akiyama K (2009). Cytokine production profile of CD4^+^ T cells from patients with active Churg-Strauss syndrome tends toward Th17. Int Arch Allergy Immunol.

[15] Schneeweis C, Rafalowicz M, Feist E, Buttgereit F, Rudolph PE, Burmester GR (2010). Increased levels of BLyS and sVCAM-1 in anti-neutrophil cytoplasmatic antibody (ANCA)-associated vasculitides (AAV). Clin Exp Rheumatol.

[16] Williams MR, Luscinskas FW (2011). Leukocyte rolling and adhesion via ICAM-1 signals to endothelial permeability. Focus on “Leukocyte rolling and adhesion both contribute to regulation of microvascular permeability to albumin via ligation of ICAM-1”. Am J Physiol Cell Physiol.

[17] Nakahigashi K, Otsuka A, Miyachi Y, Kabashima K, Tanioka M (2013). A case of Churg-Strauss syndrome: flow cytometric analysis of the surface activation markers of peripheral eosinophils. Acta Derm Venereol.

[18] Nagy JA, Benjamin L, Zeng H, Dvorak AM, Dvorak HF (2008). Vascular permeability, vascular hyperpermeability and angiogenesis. Angiogenesis.

[19] Monach PA, Tomasson G, Specks U, Stone JH, Cuthbertson D, Krischer J (2011). Circulating markers of vascular injury and angiogenesis in ANCA-associated vasculitis. Arthritis Rheum.

[20] Clarke LA, Hong Y, Eleftheriou D, Shah V, Arrigoni F, Klein NJ (2010). Endothelial injury and repair in systemic vasculitis of the young. Arthritis Rheum.

[21] Chumbley L, Harrison E, DeRemee R (1977). Allergic granulomatosis and angiitis (Churg-Strauss syndrome). Mayo Clin Proc.

[22] Pagnoux C, Mahr A, Cohen P, Guillevin L (2005). Presentation and outcome of gastrointestinal involvement in systemic necrotizing vasculitides: analysis of 62 patients with polyarteritis nodosa, microscopic polyangiitis, Wegener granulomatosis, Churg-Strauss syndrome, or rheumatoid arthritis-associated vasculitis. Medicine (Baltimore).

[23] Hokama A, Kishimoto K, Ihama Y, Kobashigawa C, Nakamoto M, Hirata T (2012). Endoscopic and radiographic features of gastrointestinal involvement in vasculitis. World J Gastrointest Endosc.

[24] Guillevin L, Lhote F, Gayraud M, Cohen P, Jarrousse B, Lortholary O (1996). Prognostic factors in polyarteritis nodosa and Churg-Strauss syndrome: a prospective study in 342 patients. Medicine (Baltimore).

[25] Cojocaru M, Cojocaru IM, Silosi I, Vrabie CD (2011). Gastrointestinal manifestations in systemic autoimmune diseases. Maedica (Buchar).

[26] Alfadda AA, Storr MA, Shaffer EA (2011). Eosinophilic colitis: epidemiology, clinical features, and current management. Therap Adv Gastroenterol.

[27] Mueller S (2008). Classification of eosinophilic gastrointestinal diseases. Best Pract Res Clin Gastroenterol.

[28] Gayraud M, Guillevin L, Toumelin P, Cohen P, Lhote F, Casassus P (2001). Long-term followup of polyarteritis nodosa, microscopic polyangiitis, and Churg-Strauss syndrome. Arthritis Rheum.

[29] Guillevin L, Pagnoux C, Seror R, Mahr A, Mouthon L, Toumelin P (2011). The five-factor score revisited. Assessment of prognosis of systemic necrotizing vasculitides based on the French Vasculitis Study Group (FVSG) Cohort. Medicine.

[30] Moosig F, Bremer JP, Hellmich B, Holle JU, Holl-Ulrich K, Laudien M (2013). A vasculitis centre based management strategy leads to improved outcome in eosinophilic granulomatosis and polyangiitis (Churg-Strauss, EGPA): monocentric experiences in 150 patients. Ann Rheum Dis.

[31] Pagnoux C, Mahr A, Cohen P, Guillevin L (2005). Presentation and outcome of gastrointestinal involvement in systemic necrotizing vasculitis. Medicine.

[32] Lucendo AJ, Arias A (2012). Eosinophilic gastroenteritis: an update. Expert Rev Gastroenterol Hepatol.

[33] DeRoche TC, Xiao SY, Liu X (2014). Histological evaluation in ulcerative colitis. Gastroenterol Rep (Oxf).

[34] Kobayashi S, Inokuma S, Setoguchi K, Kono H, Abe K (2002). Incidence of peripheral blood eosinophilia and the threshold eosinophile count for indicating hypereosinophilia-associated diseases. Allergy.

[35] Terrier B, Bièche I, Maisonobe T, Laurendeau I (2010). Interleukin-25: a cytokine linking eosinophils and adaptive immunity in Churg-Strauss syndrome. Blood.

[36] Polzer K, Karonitsch T, Neumann T (2008). Eotaxin-3 is involved in Churg-Strauss syndrome--a serum marker closely correlating with disease activity. Rheumatology (Oxford).

[37] Khoury P, Zagallo P, Talar-Williams C (2012). Serum biomarkers are similar in Churg-Strauss syndrome and hypereosinophilic syndrome. Allergy.

[38] Tsurikisawa N, Taniguchi T, Saito H, Himeno H, Ishibashi A, Suzuki S (2004). Treatment of Churg–Strauss syndrome with high-dose intravenous immunoglobulin. Ann Allerg Asthma Immunol.

[39] Tsurikisawa N, Saito H, Oshikata C, Tsuburai T, Akiyama K (2012). High-dose intravenous immunoglobulin treatment increases regulatory T cells in patients with eosinophilic granulomatosis with polyangiitis. J Rheumatol.

[40] Radder CM, Beekhuizen H, Kanhai HH, Brand A (2004). Effect of maternal anti-HPA-1a antibodies and polyclonal IVIG on the activation status of vascular endothelial cells. Clin Exp Immunol.

[41] Yoon JS, Kim HH, Han JW, Lee Y, Lee JS (2006). Effects of intravenous immunoglobulin and methylprednisolone on human umbilical vein endothelial cells in vitro. Immunobiology.

[42] Damianovich M, Blank M, Raiter A, Hardy B, Shoenfeld Y (2009). Anti-vascular endothelial growth factor (VEGF) specific activity of intravenous immunoglobulin (IVIg). Int Immunol.

[43] Barbasso Helmers S, Dastmalchi M, Alexanderson H, Nennesmo I, Esbjörnsson M, Lindvall B (2007). Limited effects of high-dose intravenous immunoglobulin (IVIG) treatment on molecular expression in muscle tissue of patients with inflammatory myopathies. Ann Rheum Dis.

[44] Masi AT, Hunder GG, Lie JT, Michel BA, Bloch DA, Arend WP (1990). The American College of Rheumatology 1990 criteria for the classification of Churg-Strauss syndrome (allergic granulomatosis and angiitis). Arthritis Rheum.

[45] National Institutes of Health. Global Strategy for Asthma Management and Prevention. National Institutes of Health National Heart, Lung and Blood Institute report. NIH publication no. 02–3659,2002:67–79.

[46] Marchand E, Cordier JF (2006). Idiopathic chronic eosinophilic pneumonia. Semin Respir Crit Care Med.

[47] Horiguchi Y, Morita Y, Tsurikisawa N, Akiyama K (2011). ^123^I-MIBG imaging detects cardiac involvement and predicts cardiac events in Churg-Strauss syndrome. Eur J Nucl Med Mol Imaging.

[48] Abril A, Calamia KT, Cohen MD (2003). The Churg–Strauss syndrome (allergic granulomatous angiitis): review and update. Semin Arthritis Rheum.

[49] Winawer SJ, Zauber AG, Fletcher RH, Stillman JS, O’Brien MJ, Levin B (2006). US Multi-Society Task Force on Colorectal Cancer; American Cancer Society. Guidelines for colonoscopy surveillance after polypectomy: a consensus update by the US Multi-Society Task Force on Colorectal Cancer and the American Cancer Society. Gastroenterology.

[50] Sanderson IR, Boyle S, Williams CB, Walker-Smith JA (1986). Histological abnormalities in biopsies from macroscopically normal colonoscopies. Arch Dis Child.

[51] Picker LJ, Singh MK, Zdraveski Z, Treer JR, Waldrop SL, Bergstresser PR (1995). Direct demonstration of cytokine synthesis heterogeneity among human memory/effector T cells by flow cytometry. Blood.

